# Waiting time for radiation therapy after breast-conserving surgery in early breast cancer: a retrospective analysis of local relapse and distant metastases in 615 patients

**DOI:** 10.1186/s40001-016-0226-9

**Published:** 2016-08-11

**Authors:** Raffaella Caponio, Maria Paola Ciliberti, Giusi Graziano, Rocco Necchia, Giovanni Scognamillo, Antonio Pascali, Sabino Bonaduce, Anna Milella, Gabriele Matichecchia, Cristian Cristofaro, Davide Di Fatta, Pasquale Tamborra, Marco Lioce

**Affiliations:** 1 U.O. Radioterapia, National Cancer Research Centre Istituto Tumori “Giovanni Paolo II”, Via O. Flacco, 65, Bari, Italy; 2 Direzione Scientifica, National Cancer Research Centre Istituto Tumori “Giovanni Paolo II”, Bari, Italy; 3 U.O. Fisica Sanitaria, National Cancer Research Centre Istituto Tumori “Giovanni Paolo II”, Bari, Italy

**Keywords:** Timing, Radiotherapy, Early-stage breast cancer, Local relapse, Distant metastases, Survival

## Abstract

**Background:**

Postoperative radiotherapy after breast-conserving surgery (BCS) is the standard in the management of breast cancer. The optimal timing for starting postoperative radiation therapy has not yet been well defined. In this study, we aimed to evaluate if the time interval between BCS and postoperative radiotherapy is related to the incidence of local and distant relapse in women with early node-negative breast cancer not receiving chemotherapy.

**Methods:**

We retrospectively analyzed clinical data concerning 615 women treated from 1984 to 2010, divided into three groups according to the timing of radiotherapy: ≤60, 61–120, and >120 days. To estimate the presence of imbalanced distribution of prognostic and treatment factors among the three groups, the *χ*2 test or the Fisher exact test were performed. Local relapse-free survival, distant metastasis-free survival (DMFS), and disease-free survival (DFS) were estimated with the Kaplan–Meier method, and multivariate Cox regression was used to test for the independent effect of timing of RT after adjusting for known confounding factors. The median follow-up time was 65.8 months.

**Results:**

Differences in distribution of age, type of hormone therapy, and year of diagnosis were statistically significant. At 15-year follow-up, we failed to detect a significant correlation between time interval and the risk of local relapse (*p* = 0.09) both at the univariate and the multivariate analysis. The DMFS and the DFS univariate analysis showed a decreased outcome when radiotherapy was started early (*p* = 0.041 and *p* = 0.046), but this was not confirmed at the multivariate analysis (*p* = 0.406 and *p* = 0.102, respectively).

**Conclusions:**

Our results show that no correlation exists between the timing of postoperative radiotherapy and the risk of local relapse or distant metastasis development in a particular subgroup of women with node-negative early breast cancer.

## Background

Breast cancer (BC) is the leading cancer among women [1] worldwide. It represented 13.5 % of all cancers in Europe in 2012 [2] and the most frequent cause of death in women. Several studies have reported a decline in BC mortality thanks to early detection and progress in cancer treatment [3, 4].

Radiotherapy (RT) after breast-conserving surgery (BCS) halves the incidence of local recurrence and reduces the cancer-specific death by a sixth. Evidence from randomized clinical trials [5–8] and meta-analyses [9, 10] demonstrated excellent local control rates and survival, equivalent to those observed with mastectomy alone.

The optimal timing for starting postoperative RT is not yet well defined. In principle, a delay between surgery and the start of RT could allow the growth of clonogenic cells in the tumor bed and the development of radioresistance [11]. Delays of >8–12 weeks seem to increase the risk of local relapse in observational studies, but results are conflicting. Moreover, no phase III studies about the optimal interval between surgery and radiotherapy are available.

National Canadian clinical practice guidelines recommend that RT should be given <12 weeks after BCS to keep the incidence of local failure and disease-free survival (DFS) similar to that of mastectomy [12]. The National Cancer Intelligence Network suggests that “the time between surgery and the start of radiotherapy should be no more than 31 days” [13]. The Merseyside and Cheshire cancer network guidelines report that “radiotherapy should be started within 12 weeks of the date of surgery” [14]. The latest Italian guidelines [15] recommend starting RT earlier than 20 weeks after surgery if no systemic treatment is given, especially in women <40 years of age and/or with positive margins [16, 17].

In this study, we retrospectively analyzed the long-term follow-up of 615 women treated with BCS and whole breast conventional radiation therapy (WBI) for early BC. The aim was to investigate the relationship between waiting time for postoperative RT and the development of local relapse and distant metastases.

## Methods

We analyzed data concerning 615 patients with early BC who underwent WBI with conventional fractionation at our institution between December 1984 and December 2010.

All patients had DCIS-T1-T2, N0, M0 BC, and underwent BCS (quadrantectomy ± sentinel lymph node biopsy and/or axillary dissection). After surgery, they all received WBI using an isocentric technique with two tangential fields, followed by a boost on the tumor bed in 89.6 % of cases. The mean dose was 50 Gy (range 40–60 Gy) for WBI, delivered in 2 Gy fractions five times a week. The dose was prescribed at the isocenter, on the basis of the ICRU 50 guidelines [18], and the CTV (clinical target volume) was set at a 95 % isodose level. The dose to the breast was administered with a ≥6 MV photon beam; the tumor bed boost was administrated by electrons or photon beam to a total dose of 10 Gy in the case of negative surgical margins (96.7 %) and to higher doses (14–20 Gy) in the case of close or positive margins (3.3 %). Surgical margins were considered free if ≥2 mm, close if <2 mm, and positive if disease persisted on the margin. Patients with close or positive margins refused reexcision or did not receive this recommendation by the surgeon.

No patient received chemotherapy. Hormone therapy (HT) was prescribed in 80.2 % of patients (40.7 % aromatase inhibitors, 25.5 % tamoxifen, and 13.8 % tamoxifen plus gonadotropine-releasing hormon analog). Almost all patients started endocrine therapy before radiotherapy.

Patients without positivity of hormone receptors did not receive chemotherapy for age or comorbidities.

The waiting list for BC patients was not formally conditioned by protocols or guidelines concerning risk factors. The time of delay was mostly conditioned by the delay in referring to the radiotherapy center and the overall waiting list for starting radiotherapy.

After RT, patients were evaluated every 4 months for the first 2 years, then every 6 months until the 5th year and henceforth every year.

Eighteen women (2.9 %) developed a contralateral metachronous epithelial BC.

One patient developed a cutaneous angiosarcoma at the level of the breast surgical scar, probably related to previous breast irradiation, 9 years after treatment.

All patient subscribed a written consent to treatment. This analysis was approved by our institutional Ethics Committee.

### Statistical methods

For the survival analysis, the BCS date, defined as the date of the last surgery on the breast, was used as the start of observation, and the date of the last medical follow-up visit was used as the end of the follow-up period. Timing of RT was calculated as the interval between BCS and the RT start date, defined as the date in which the first fraction of RT was administered.

Patients were categorized into three groups according to timing of RT (T1: <60 days; T2: 60–120 days; T3: >120 days).

The *χ*2 test or the Fisher exact test, when appropriate, was used to calculate intergroup differences of some clinical categorical variables. Results are shown in Table 1.

**Table 1:** Distribution of patients on the basis of clinicopathological features and time delay

Variable	*N*	≤60 days	*N*	60–120 days	*N*	>120 days	*p* value
N. group		53		298		264	
Age							
≤50	53	31 (58.49)	298	85 (28.52)	264	56 (21.21)	<0.0001
>50	53	22 (41.51)	298	213 (71.48)	264	208 (78.79)	
Grading							
G1	32	9 (28.13)	228	63 (27.63)	219	71 (32.42)	0.741
G2	32	20 (62.50)	228	132 (57.89)	219	118 (53.88)	
G3	32	3 (9.38)	228	33 (14.47)	219	30 (13.70)	
Hystologic type							
Ductal infiltrating	52	39 (75.00)	297	225 (75.76)	264	176 (66.67)	0.2576
Lobular infiltrating	52	5 (9.62)	297	16 (5.39)	264	21 (7.95)	
Ductal in situ	52	4 (7.69)	297	32 (10.77)	264	35 (13.26)	
Other	52	4 (7.69)	297	24 (8.08)	264	32 (12.12)	
Margins							
Negative	53	52 (98.11)	298	292 (97.99)	264	251 (95.08)	0.0716
Close	53	0 (0.00)	298	3 (1.01)	264	1 (0.38)	
Positive	53	1 (1.89)	298	3 (1.01)	264	12 (4.55)	
Hormone therapy							
No	34	3 (8.82)	258	14 (5.43)	239	21 (8.79)	0.323
Yes	34	31 (91.18)	258	244 (94.57)	239	218 (91.21)	
Estrogen receptor status							
Negative	35	2 (5.71)	267	14 (5.24)	248	17 (6.85)	0.7418
Positive	35	33 (94.29)	267	253 (94.76)	248	231 (93.15)	
Site							
Right breast	53	24 (45.28)	291	140 (48.11)	257	139 (54.09)	0.2778
Left breast	53	29 (54.72)	291	151 (51.89)	257	118 (45.91)	
T stage							
DCIS	53	5 (9.43)	298	44 (14.77)	264	52 (19.70)	0.2187
T1	53	38 (71.70)	298	216 (72.48)	264	180 (68.18)	
T2	53	10 (18.87)	298	38 (12.75)	264	32 (12.12)	
Hormone therapy type							
None	33	3 (9.09)	258	14 (5.43)	239	21 (8.79)	0.0006
AI	33	5 (15.15)	258	117 (45.35)	239	128 (53.56)	
Tamoxifen	33	14 (42.42)	258	85 (32.95)	239	58 (24.27)	
LHRH + tamoxifen	33	11 (33.33)	258	42 (16.28)	239	32 (13.39)	
Year of diagnosis							
1980–1995	53	16 (30.19)	298	12 (4.03)	264	3 (1.14)	<0.0001
1996–2010	53	37 (69.81)	298	286 (95.97)	264	61 (98.86)	

*N* number of patients; *AI* aromatase Inhibitors; *LHRH* luteinizing hormone-releasing hormone agonists

Survival statistics (Local relapse-free survival—LRFS, distant metastasis-free survival—DMFS, and disease-free survival-DFS) were estimated with the Kaplan–Meier method, and differences between groups were validated by the Log-rank test. The multivariate Cox regression was used to test for the independent effect of timing of RT after adjusting for known confounding factors. The results were presented as hazard ratios (HR) with corresponding 95 % confidence intervals. The group with the shortest time interval, <60 days, was the reference category. Statistical significance was achieved at a *p* < 0.05.

All the analyses were performed using the Statistical Analysis System (SAS Institute, Cary, NC) software.

## Results

The median time of follow-up, defined as the median time between BCS and last follow-up, was 65.8 months (range 4–179); only 7.5 % of patients had a follow-up <6 months. The median waiting time from surgery to the start of RT was 111 days (range 21–532 days).

The mean patient age was 58 years (range 21–87).

Differences in distribution of age, type of hormone therapy (HT), and year of diagnosis among the three groups were statistically significant (*p* < 0.0001, *p* = 0.0006 and *p* < 0.0001, respectively) (Table 1).

### Local relapse-free survival

Overall, we found 11 disease relapses in the treated breast (1.8 %): four of them (36.4 %) in the first group, 4 (36.4 %) in the second group, and 3 (27.3 %) in the third group.

The median time between surgery and the occurrence of local relapse was 81.7 months (range 16.6–230.4).

**Fig. 1:**
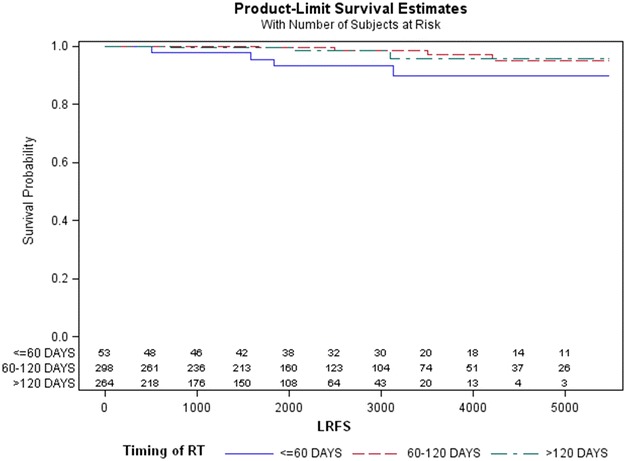
Kaplan–Meier estimates of local relapse-free survival (LRFS) related to time interval between surgery and radiotherapy (*p* = 0.09)

The Kaplan–Meier method found a LRFS rate of 89.7 % for the first group (95 % CI 0.74–0.96), 95.2 % for the second (95 % CI 0.86–0.98), and 95.8 % for the third group (95 % CI 0.84–0.98). No statistically significant relationship between local relapse and timing of RT was found (*p* = 0.09) (Fig. 1).

When the significant patient variables (age, type of HT, and year of diagnosis) shown in Table 1 were considered in the analysis, the HR for the second and third group were 0.28 (95 % CI 0.05–1.58) and 0.58 (95 % CI 0.09–3.62), respectively, compared with the first group, as shown in Table 2. The *p* value was 0.338.

**Table 2:** Adjusted proportional hazard regression results

Timing of RT	HR (95 % CI)	*p*
LRFS (days)		
<60	1	0.338
61–120	0.28 (0.53–1.58)	
>120	0.58 (0.09–3.62)	
DMFS (days)		
<60	1	0.406
61–120	0.32 (0.06–1.70)	
>120	–	
DFS (days)		
<60	1	0.102
61–120	0.36 (0.10–1.24)	
>120	0.18 (0.04–0.91)	

*HR* hazard risk; *LRFS* local relapse-free survival; *DMSF* distant metastasis-free survival; *DFS* disease-free survival

### Distant metastasis-free survival

In our study, 11 patients (1.8 %) presented distant metastases: 3 (27.3 %) in the group with a delay ≤60 days and 8 (72.7 %) in the group with a delay of 61–120 days. No metastases were found in the third group with a longer time delay.

The median time between surgery and the occurrence of local relapse was 44.6 months (range 14.4–124.7).

The Kaplan–Meier method found a DMFS rate of 91.3 % for the first group (95 % CI 0.73–0.97), 95.2 % for the second group (95 % CI 0.90–0.97), and 100 % for the third group.

**Fig. 2:**
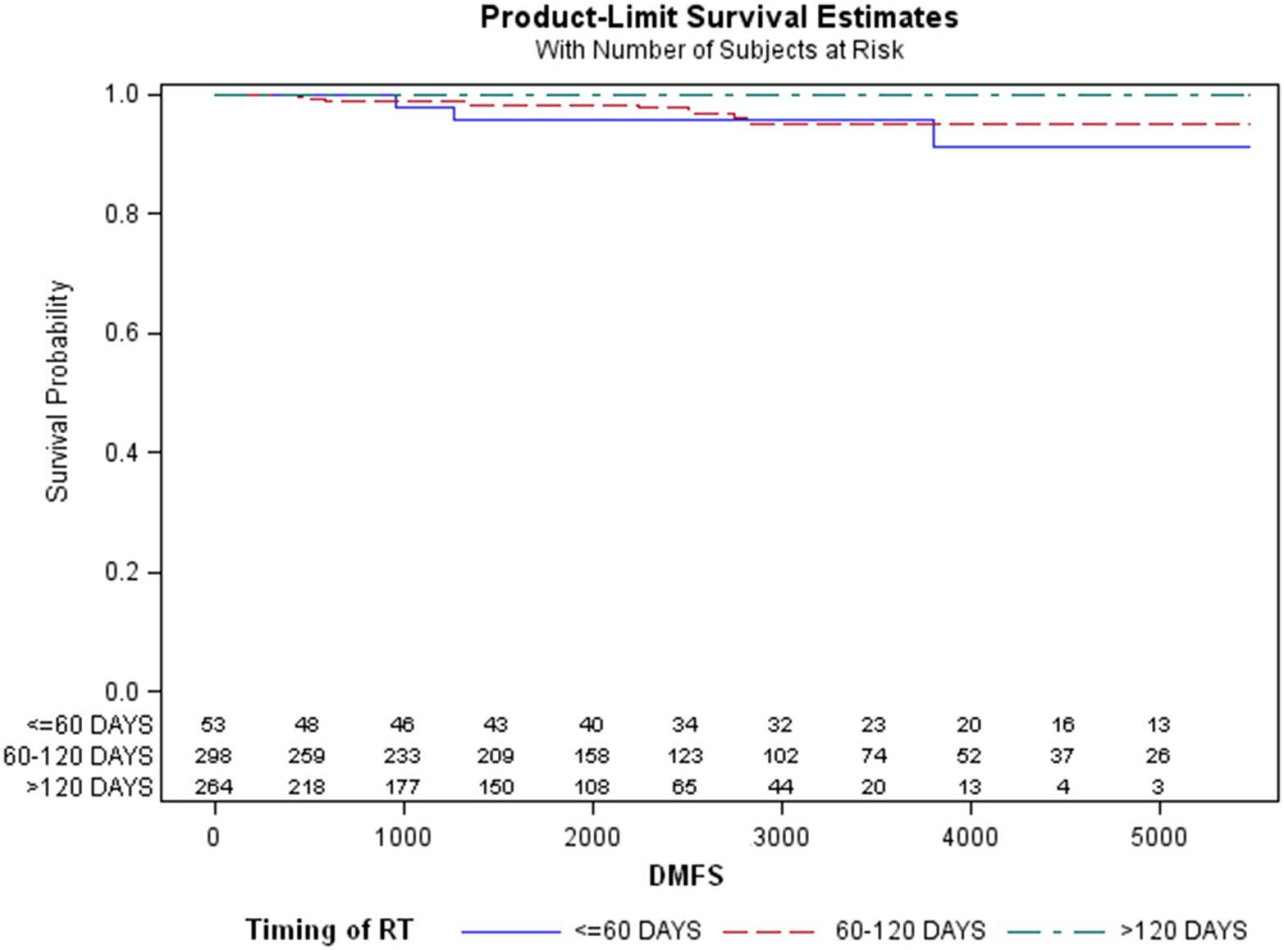
Kaplan–Meier estimates of distant metastasis-free survival (DMFS) related to time interval between surgery and radiotherapy (*p* = 0.041)

At the univariate analysis, we found a statistically significant difference in the distribution of metastases among the three groups (*p* = 0.041) (Fig. 2).

Nevertheless, when corrected for age, type of HT, and year of diagnosis, the HR of the second group was 0.32 (95 % CI 0.06–1.70). It was not possible to calculate the HR for the third group (>120 days) because no metastases occurred in this group (Table 2). The *p* value was 0.406.

### Disease-free survival

Twenty-one patients (3.4 %) presented a failure: one patient had both local and distant relapse of disease. Six disease relapses occurred in the first group (28.6 %), 12 (57.1 %) in the second group and 3 (14.3 %) in the third group.

The median time between surgery and a disease-related event, recurrence or metastases, was 67.4 months (range 14.4–230.4).

**Fig. 3:**
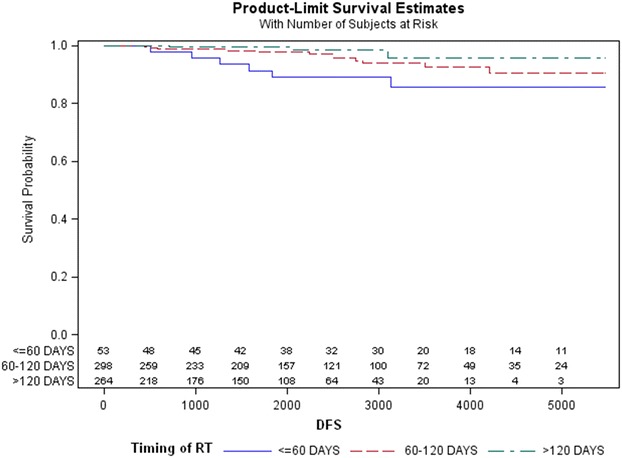
Kaplan–Meier estimates of disease-free survival (DFS) related to time interval between surgery and radiotherapy (*p* = 0.046)

The DFS rate calculated with the Kaplan–Meier method was 85.7 % (95 % CI 0.70–0.93) for the first group, 90.5 % for the second (95 % CI 0.82–0.95), and 95.8 % for the third group (95 % CI 0.85–0.98). This difference was marginally statistically significant (*p* = 0.046) (Fig. 3).

Multivariate analysis confirmed that timing of RT is not an independent prognostic factor (*p* = 0.102) (Table 2). The HRs for the second and third group were 0.36 (95 % CI 0.10–1.24) and 0.18 (95 % CI 0.04–0.91), respectively, compared with the first group.

An analysis aiming to find correlation between local relapse or distant metastases and certain characteristics, as patient age, status of surgical margins or tumor histology, grade, and stage, was conducted, but no statistically significant differences were found among these subgroups.

## Discussion

The interval between BCS and postoperative RT in breast cancer treatment can significantly change. Causes of this variation could be patient compliance and socioeconomic status; geographic distribution of radiotherapy centers; long waiting lists; characteristics of patients and their cancer (age, prognostic factors, presence of comorbidities, etc.) [19], and surgical complications (slow wound healing, inflammation, or infections). Moreover, the waiting time for radiotherapy has increased dramatically during the last decades [20, 21] due to the increased demand for radiation treatments.

In theory, an excessive time to RT could be associated with an increased risk of local recurrence and with a subsequent increased risk of metastasis. Some radiobiological models [22] have shown a small decrease in local control of 1–2 % per month’s delay in treatment.

However, clinical data are not always consistent with this theory. No phase III trials have been published, but only several retrospective analyses: some experiences showed a positive association, while others did not find a clear correlation.

One of the first studies on the relationship between waiting time for radiotherapy and clinical outcomes was conducted in 1985 at the Institut Gustave Roussy in France by Clarke et al [23]. They reviewed 436 patients with T1 and small T2 breast carcinoma, finding a significant increase in local relapse in the group that received radiotherapy more than 7 weeks after surgery. These results were not confirmed on multivariate analysis.

One of the largest studies published reviewed data concerning 13,907 women ≥65 years with stage I–II BC diagnosed and treated between 1991 and 1999 and not receiving chemotherapy, extracted from the surveillance, epidemiology, and end results (SEER) registry [24]. The Authors concluded that “patients who do not receive RT until more than 12 weeks after BCS appear to have poorer survival.” A successive SEER database analysis conducted in 2010 [25] showed that intervals over 6 weeks were associated with an increased incidence of local recurrence.

Many other retrospective studies found a significant correlation between a long RT delay and increased rate of local relapse, with or without impact on survival, in the same cohort of patients [16, 26–28]. Simultaneously, another group of retrospective analyses conducted on similar patients failed to find a significant variation of these endpoints [29–34].

Contrary to all this evidence, a recent retrospective analysis [35] on 1107 women with early-stage BC without lymph node metastases or adjuvant systemic therapies found a significantly decreased DMFS and disease-specific survival in the tertile that started radiotherapy early after BCS (<45 days) compared with the other two tertiles (45–56 and 57–112 days), without differences in local control. The Authors attributed these results to residual confounding factors (age and prognostic factors) that could lead the physician to start radiotherapy early in high-risk patients. Another probability is that starting radiotherapy too early could induce vascular damage, a delay in stromal cell growth, and an increase in tumor cell damage: these mechanisms could lead to a easier spread of metastatic cells, as suggested in a previous study on radiation induced metastatic spread and angiogenesis.

Further information on the optimal timing of RT in early-stage cancer treated with sole Radiotherapy could be extracted from studies that also included patients with advanced-stage BC, through the analysis of subgroups. Even in these studies results are not unequivocal, ranging from the nonexistence of association [17, 36, 37] to a mild [38] or significant [39–45] correlation between RT delay and local recurrence rate.

Some Authors have tried to summarize these conflicting results through meta-analysis and reviews, but final analysis remains conflicting.

The critical review conducted by Hebert-Croteau published between 1985 and 2000 reported a nonsignificant association between time interval and the risk of local recurrence or death related to BC [46]. In contrast, a meta-analysis by Huang et al [47] published in 2003 and including 7401 BC patients showed a 1.62-fold increase in local recurrence rate when radiotherapy was administered >8 weeks after BCS. In another review, Ruo Redda et al. [48] suggested that, in the group of patients that do not need any treatments other than RT, the time interval should not exceed 8 weeks. A more recent systematic review conducted by Chen et al. [49] evaluated time interval to RT as a continuous variable, showing an increase of the RR of local recurrence of 1.11 every month after BCS. An association between waiting time and distant metastases or overall survival did not emerge. The most recent systematic review by Tsoutsou et al. [50] found that an interval of more than 8–12 weeks increased local recurrence rates when RT was administered as the sole adjuvant modality.

In our study, we aimed to explore the correlation between the delay of postoperative RT and the development of local recurrence and distant metastases in women with node-negative T1–T2 BC treated with RT, with/without HT but without chemotherapy. We divided the 615 patients into three subgroups according to the timing of RT (T1: <60 days; T2: 60–120 days; T3: >120 days). The majority of patients were in the second group.

Our experience failed to detect a significant correlation between BCS-to-RT time interval and the risk of local relapse in early-stage BC patients (*p* = 0.09). This lack of significance was confirmed at the multivariate analysis adjusted for age and type of HT, indicating that the failure to find a univariate relationship between timing of RT and BC local recurrence was not imputable to an uneven distribution of these two variables.

On the other hand, we found an unexpected but significant inverse correlation at the univariate analysis between timing of RT and the risk of distant metastases development. In particular, at the Kaplan–Meier analysis, the DMFS was 91.3 % for the group that received RT prior to 60 days, 95.2 % for the group who received RT between 61 and 120 days after surgery, and 100 % for the group who received RT more than 120 days after BCS (*p* = 0.041). The outcome seemed to be worse in patients who started RT early (<60 days after surgery). However, this correlation was not confirmed at the multivariate analysis (HR T2: 0.32; 95 % CI 0.06–1.70, *p* = 0.406) when age of patients, type of HT, and year of diagnosis were considered.

Similarly, the DFS seemed to be correlated to timing of RT at the univariate analysis with a marginal statistical significance, (T1: 85.7 %, T2: 90.5 %; T3: 95.8 %; *p* = 0.046), apparently confirming the worse outcome of patients in the first group. Also in this case, any correlation was lost when analysis was adjusted for known confounding factors (HR T2: 0.36; 95 % CI 0.10–1.24; HR T3: 0.18, 95 % CI 0.04–0.91, *p* = 0.102).

These results seem to be related to an unbalanced distribution of two main variables (age and year of diagnosis) among the three groups. In turn, the third confounding variable (type of HT) is often strictly dependent on the age of patients. Probably, these factors operated as real confounding factors when DMFS and overall DFS are considered. In particular, we observed a prevalence of young women, with a consequent younger median age, in the first group compared with the other two groups. It is possible that physicians involuntarily expedited RT for cases they perceived to be at higher risk of local or distant recurrence, such as young women. The correlation between age and rate of local [5, 10, 51] and distant [52] relapse, especially visceral metastases [53], has been demonstrated in several large studies and is often considered by physicians during the first evaluation of a breast cancer patient.

Moreover, in the first group, we found a greater proportion of patients treated in the 1980s and early 90s compared with the other two groups, probably due to the lesser impact of waiting lists in past years. This finding could justify the worse outcome in terms of DMFS and DFS which emerged for the T1 group at the univariate analysis: when these BC patients were treated, RT technology was not advanced: this could lead to an under-dosage or a partial miss of the radiotherapy target. Moreover, in past decades, accurate guidelines about systemic treatment of BC were not available: nowadays, adjuvant chemotherapy is more often prescribed [54], so it is possible that patients treated in the first years of our analysis would have to be treated with more aggressive therapies because they presented high-risk features.

Finally, we know that local and distant relapse usually occur after several years: patients in the T1 group, treated in the 80s and early 90s, are more likely to develop relapses because of their longer follow-up rather than because of a real correlation with timing of RT. All these confounding factors, considered in the multivariate analysis, could explain the lack of significant correlation between timing of RT and all the events considered.

Evidently, our study has some limits intrinsic to its retrospective nature, such as the bias regarding patient selection and their unequal distribution among the three timing groups.

Moreover, although we followed patients for a long time (until 15 years), the median follow-up is only about 6 years, because of the large time of accrual and the presence of patients who did not attended the follow-up program. Local recurrence in BC could develop also 10–15 years after BCS, so a longer follow-up is requested to obtain more realistic data.

## Conclusions

Our results showed that there is no correlation between the BCS-to-RT interval time and the risk of local relapse or distant metastases in a particular subset of node-negative early-stage BC patients not receiving chemotherapy. However, our results are limited by the retrospective nature of the study, so they should be validated by randomized studies or well selected meta-analysis, with the aim of filling the gap in clinical evidence about the optimal time interval between BCS and PORT.

## Abbreviations

BC: breast cancer
BCS: breast-conserving surgery
RT: radiotherapy
LRFS: local relapse-free survival
DMFS: distant metastasis-free survival
DFS: disease-free survival
HT: hormone therapy
CI: confidence interval
HR: hazard ratio
